# 2,4,6-Trimethyl-1,3,5-tris­(morpholino­meth­yl)benzene

**DOI:** 10.1107/S1600536808008763

**Published:** 2008-05-03

**Authors:** Hong-Ji Ma, Chen Xu, Zhi-Qiang Wang, Le Zhou, Bao-Ming Ji

**Affiliations:** a Northwest Agriculture and Forestry University, Yangling 712100, People’s Republic of China; b College of Chemistry and Chemical Engineering, Luoyang Normal University, Luoyang 471022, People’s Republic of China

## Abstract

In the title compound, C_24_H_39_N_3_O_3_, the H atoms of the methyl groups are disordered over two positions, with site-occupation factors fixed at 0.5. The three morpholino groups are arranged in an asymmetrical fashion with respect to the anchoring mesitylene ring and adopt chair conformations. Inter­molecular C—H⋯π inter­actions link the mol­ecules into a one-dimensional chain structure.

## Related literature

For related literature, see: Blackman (2005 ▶); Nakai *et al.* (2003 ▶); Van der Made & Van der Made (1993 ▶); Zeng & Zimmerman (1997 ▶).
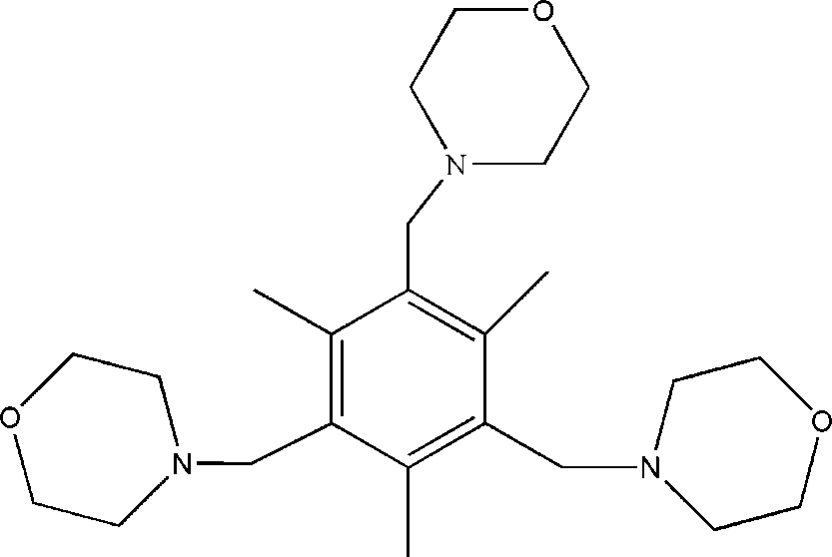


## Experimental

### 

#### Crystal data

C_24_H_39_N_3_O_3_
*M* *_r_* = 417.58
Monoclinic, 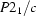

*a* = 11.0139 (10) Å
*b* = 24.131 (2) Å
*c* = 9.2941 (8) Å
β = 108.2330 (10)°
*V* = 2346.2 (4) Å^3^
*Z* = 4
Mo *K*α radiation
μ = 0.08 mm^−1^
*T* = 291 (2) K
0.49 × 0.37 × 0.34 mm

#### Data collection

Bruker SMART APEXII CCD area-detector diffractometer
Absorption correction: multi-scan (*SADABS*; Sheldrick, 1996 ▶) *T*_min_ = 0.963, *T*_max_ = 0.974
16860 measured reflections
4350 independent reflections
3409 reflections with *I* > 2σ(*I*)
*R*_int_ = 0.019

#### Refinement

*R*[*F* ^2^ > 2σ(*F* ^2^)] = 0.044
*wR*(*F* ^2^) = 0.125
*S* = 1.03
4350 reflections
271 parameters
H-atom parameters constrained
Δρ_max_ = 0.20 e Å^−3^
Δρ_min_ = −0.17 e Å^−3^

### 

Data collection: *APEX2* (Bruker, 2007 ▶); cell refinement: *APEX2*; data reduction: *SAINT* (Bruker, 2007 ▶); program(s) used to solve structure: *SHELXS97* (Sheldrick, 2008 ▶); program(s) used to refine structure: *SHELXL97* (Sheldrick, 2008 ▶); molecular graphics: *SHELXTL* (Sheldrick, 2008 ▶); software used to prepare material for publication: *SHELXTL*.

## Supplementary Material

Crystal structure: contains datablocks global, I. DOI: 10.1107/S1600536808008763/fj2099sup1.cif

Structure factors: contains datablocks I. DOI: 10.1107/S1600536808008763/fj2099Isup2.hkl

Additional supplementary materials:  crystallographic information; 3D view; checkCIF report

## Figures and Tables

**Table 1:** Hydrogen-bond geometry (Å, °)

*D*—H⋯*A*	*D*—H	H⋯*A*	*D*⋯*A*	*D*—H⋯*A*
C11—H11*B*⋯*Cg*1^i^	0.97	2.90	3.731 (2)	144
C7—H7*B*⋯*Cg*1^ii^	0.97	2.80	3.528 (2)	132

Symmetry codes: (i) 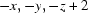; (ii) 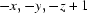. *Cg*1 is the centroid of the benzene ring.

**Fig. 1.**
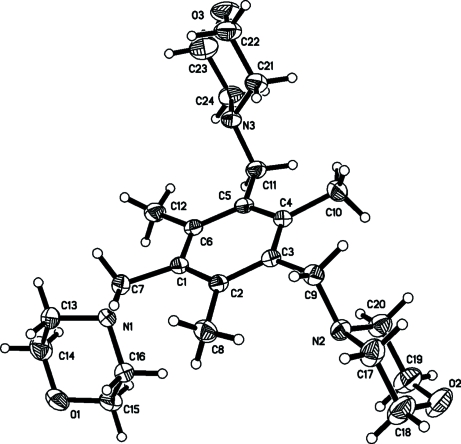
The molecular structure of the title compound shown using 30% probability ellipsoids. Only one position of the disordered methyl hydrogen atoms are shown.

**Fig. 2.**
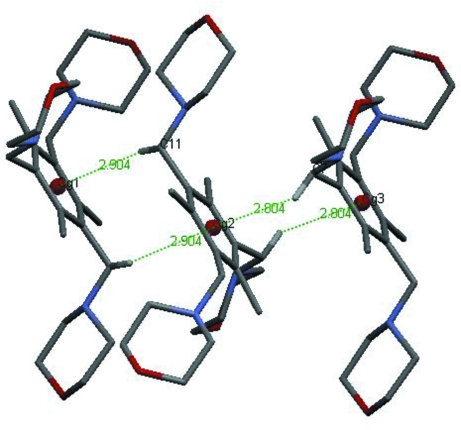
Partial view of the crystal packing showing the formation of the infinite chain of molecules formed by the CH-π interactions. H atoms not involved in CH-π interactions have been omitted for clarity.

## supplementary crystallographic information

### Comment

Tripodal ligands based on nitrogen heterocycles have been widely employed in many areas of inorganic chemistry(Blackman, 2005). For example, tripodal ligands with an arene core have been found to be one of the most useful organic building blocks in construction of metal-organic frameworks(MOFs)(Zeng, *et al.*, 1997). Herein we report the synthesis,characterization and crystal structure of the title tripodal ligand.

A view of the molecular structure of the title compound is given in Fig.1. A l l the bond distances and angles are within normal ranges, the *C*(morpholino-1-ylmethyl)-N distances [1.4669 (19)- 1.4676 (19) Å] are similar to those of the related complex [1.464 (2)- 1.467 (2) Å] (Nakai, *et al.*, 2003). The *C*(methyl and morpholino-1-ylmethyl)atoms and benzene ring are approximately coplanar, the three morpholino groups are arranged in an asymmetrical fashion with respect to the anchoring mesitylene ring and adopt chair conformations. Fig. 2 shows that in the crystal there exist two types of intermolecular CH-π interactions [H11B—*Cg*(-x, -y, 2-z) = 2.804Å and H7B—*Cg*(-x, -y, 1-z) = 2.904 Å; *Cg* is the centroid of the benzene ring], which are attributed to construct the one-dimension chain structure of the title compound.

### Experimental

1,3,5-tris(bromomethyl)-2,4,6-trimethylbenzene was synthesized according to the reported procedurewas (Van der Made, *et al.*, 1993). Morpholine (9 mmol) and NaH (27 mmol) were dissolved in dry dioxane (25 ml) and the solution was stirred for 2 h at room temperature, then 1,3,5-tris(bromomethyl)-2,4,6- trimethylbenzene (3 mmol)was added.The resultant solution was heated to reflux for 6 h, removal of solvent resulted in a white powder that was recrystallized from dichloromethane-petroleum ether solution at room temperature to give the desired product as colorless crystals suitable for single-crystal X-ray diffraction (yield 65%; m.p 410–412 K). Analysis found: C 69.15, H 9.22, N 10.25%; requires: C 69.03, H 9.41, N 10.06%. IR data (v_max/ cm^-1^): 2851, 2804, 1452, 1345, 1115, 998, 907, 863. NMR δ(H) 2.43(9*H*,s), 2.46(12*H*,s), 3.55(6*H*,s), 3.63(12*H*,s). MS-ESI^+^ [m/*z*]: 418.4(*M*+H).

### Refinement

H atoms were positioned geometrically and treated as riding, with C—H bond lengths constrained to 0.93 (aromatic CH), or 0.96 Å (methyl CH_3_), and O—H = 0.82 Å, N—H = 0.86 Å, and with *U*_iso_(H) = 1.2Ueq (CH or NH) or *U*_iso_(H) = 1.5Ueq(CH_3_ or OH). The hydrogen atoms of methyl groups are disordered over two positions, with a 1:1 occupancy ratio.

### Crystal data

**Table:**

C_24_H_39_N_3_O_3_	*F*_000_ = 912
*M**_r_* = 417.58	*D*_x_ = 1.182 Mg m^−^^3^
Monoclinic, *P*2_1_/*c*	Mo *K*α radiation λ = 0.71073 Å
*a* = 11.0139 (10) Å	Cell parameters from 6077 reflections
*b* = 24.131 (2) Å	θ = 2.5–28.1º
*c* = 9.2941 (8) Å	µ = 0.08 mm^−^^1^
β = 108.2330 (10)º	*T* = 291 (2) K
*V* = 2346.2 (4) Å^3^	Block, colourless
*Z* = 4	0.49 × 0.37 × 0.34 mm

### Data collection

**Table:**

Bruker SMART APEXII CCD area-detector diffractometer	4350 independent reflections
Radiation source: fine-focus sealed tube	3409 reflections with *I* > 2σ(*I*)
Monochromator: graphite	*R*_int_ = 0.019
*T* = 291(2) K	θ_max_ = 25.5º
φ and ω scans	θ_min_ = 2.5º
Absorption correction: multi-scan(SADABS; Sheldrick, 1996)	*h* = −13→13
*T*_min_ = 0.963, *T*_max_ = 0.974	*k* = −29→28
16860 measured reflections	*l* = −11→11

### Refinement

**Table:**

Refinement on *F*^2^	Secondary atom site location: difference Fourier map
Least-squares matrix: full	Hydrogen site location: inferred from neighbouring sites
*R*[*F*^2^ > 2σ(*F*^2^)] = 0.044	H-atom parameters constrained
*wR*(*F*^2^) = 0.125	*w* = 1/[σ^2^(*F*_o_^2^) + (0.061*P*)^2^ + 0.5317*P*] where *P* = (*F*_o_^2^ + 2*F*_c_^2^)/3
*S* = 1.03	(Δ/σ)_max_ < 0.001
4350 reflections	Δρ_max_ = 0.20 e Å^−^^3^
271 parameters	Δρ_min_ = −0.16 e Å^−^^3^
Primary atom site location: structure-invariant direct methods	Extinction correction: none

### Special details

**Table:**

Geometry. All e.s.d.'s (except the e.s.d. in the dihedral angle between two l.s. planes) are estimated using the full covariance matrix. The cell e.s.d.'s are taken into account individually in the estimation of e.s.d.'s in distances, angles and torsion angles; correlations between e.s.d.'s in cell parameters are only used when they are defined by crystal symmetry. An approximate (isotropic) treatment of cell e.s.d.'s is used for estimating e.s.d.'s involving l.s. planes.
Refinement. Refinement of *F*^2^ against ALL reflections. The weighted *R*-factor *wR* and goodness of fit *S* are based on *F*^2^, conventional *R*-factors *R* are based on *F*, with *F* set to zero for negative *F*^2^. The threshold expression of *F*^2^ > σ(*F*^2^) is used only for calculating *R*-factors(gt) *etc*. and is not relevant to the choice of reflections for refinement. *R*-factors based on *F*^2^ are statistically about twice as large as those based on *F*, and *R*- factors based on ALL data will be even larger.

### Fractional atomic coordinates and isotropic or equivalent isotropic displacement parameters (Å^2^)

**Table:**

	*x*	*y*	*z*	*U*_iso_*/*U*_eq_	Occ. (<1)
O1	−0.47311 (11)	0.13824 (5)	0.54089 (16)	0.0666 (4)	
O2	0.46925 (15)	0.21500 (6)	1.12778 (16)	0.0820 (4)	
O3	0.11437 (19)	−0.24231 (5)	0.84017 (19)	0.0984 (6)	
N1	−0.25289 (11)	0.07200 (5)	0.56752 (14)	0.0428 (3)	
N2	0.33067 (12)	0.13735 (5)	0.90381 (14)	0.0437 (3)	
N3	0.06308 (13)	−0.12834 (5)	0.87425 (15)	0.0481 (3)	
C1	−0.02514 (13)	0.03908 (6)	0.65905 (15)	0.0377 (3)	
C2	0.08664 (13)	0.07108 (5)	0.69121 (16)	0.0381 (3)	
C3	0.19919 (13)	0.05277 (5)	0.80117 (16)	0.0373 (3)	
C4	0.19841 (13)	0.00444 (5)	0.88471 (16)	0.0391 (3)	
C5	0.08374 (13)	−0.02549 (5)	0.85949 (16)	0.0380 (3)	
C6	−0.02704 (13)	−0.00860 (6)	0.74496 (16)	0.0383 (3)	
C7	−0.14249 (14)	0.05271 (6)	0.52502 (16)	0.0449 (4)	
H7A	−0.1197	0.0811	0.4642	0.054*	
H7B	−0.1671	0.0199	0.4624	0.054*	
C8	0.08791 (17)	0.12500 (7)	0.6081 (2)	0.0544 (4)	
H8A	0.1715	0.1413	0.6443	0.082*	0.50
H8B	0.0264	0.1500	0.6259	0.082*	0.50
H8C	0.0666	0.1178	0.5015	0.082*	0.50
H8D	0.0048	0.1315	0.5368	0.082*	0.50
H8E	0.1499	0.1227	0.5552	0.082*	0.50
H8F	0.1098	0.1549	0.6796	0.082*	0.50
C9	0.32124 (14)	0.08548 (6)	0.81870 (18)	0.0441 (4)	
H9A	0.3940	0.0623	0.8696	0.053*	
H9B	0.3263	0.0940	0.7187	0.053*	
C10	0.32062 (15)	−0.01643 (7)	0.9995 (2)	0.0551 (4)	
H10A	0.3029	−0.0497	1.0460	0.083*	0.50
H10B	0.3533	0.0113	1.0759	0.083*	0.50
H10C	0.3829	−0.0241	0.9494	0.083*	0.50
H10D	0.3898	0.0081	1.0015	0.083*	0.50
H10E	0.3394	−0.0530	0.9716	0.083*	0.50
H10F	0.3099	−0.0176	1.0981	0.083*	0.50
C11	0.07458 (16)	−0.07542 (6)	0.95527 (18)	0.0463 (4)	
H11A	0.1501	−0.0766	1.0437	0.056*	
H11B	0.0009	−0.0709	0.9903	0.056*	
C12	−0.14914 (15)	−0.04149 (6)	0.7153 (2)	0.0541 (4)	
H12A	−0.1345	−0.0726	0.7831	0.081*	0.50
H12B	−0.1761	−0.0545	0.6126	0.081*	0.50
H12C	−0.2145	−0.0183	0.7315	0.081*	0.50
H12D	−0.2155	−0.0243	0.6350	0.081*	0.50
H12E	−0.1739	−0.0424	0.8055	0.081*	0.50
H12F	−0.1355	−0.0786	0.6866	0.081*	0.50
C13	−0.37183 (15)	0.06447 (7)	0.4433 (2)	0.0557 (4)	
H13A	−0.3815	0.0259	0.4127	0.067*	
H13B	−0.3700	0.0866	0.3569	0.067*	
C14	−0.48270 (17)	0.08214 (8)	0.4958 (3)	0.0687 (5)	
H14A	−0.5620	0.0766	0.4142	0.082*	
H14B	−0.4852	0.0590	0.5803	0.082*	
C15	−0.35579 (17)	0.14720 (8)	0.6582 (2)	0.0615 (5)	
H15A	−0.3560	0.1262	0.7471	0.074*	
H15B	−0.3483	0.1862	0.6854	0.074*	
C16	−0.24239 (15)	0.13020 (6)	0.61083 (19)	0.0481 (4)	
H16A	−0.2385	0.1528	0.5260	0.058*	
H16B	−0.1643	0.1361	0.6940	0.058*	
C17	0.43157 (18)	0.17266 (7)	0.8820 (2)	0.0610 (5)	
H17A	0.4125	0.1811	0.7752	0.073*	
H17B	0.5125	0.1530	0.9151	0.073*	
C18	0.4424 (2)	0.22552 (8)	0.9701 (2)	0.0802 (6)	
H18A	0.5100	0.2481	0.9546	0.096*	
H18B	0.3629	0.2460	0.9329	0.096*	
C19	0.3719 (2)	0.18151 (8)	1.1505 (2)	0.0746 (6)	
H19A	0.2914	0.2014	1.1171	0.090*	
H19B	0.3913	0.1739	1.2577	0.090*	
C20	0.35830 (18)	0.12758 (7)	1.06520 (19)	0.0548 (4)	
H20A	0.4368	0.1065	1.1029	0.066*	
H20B	0.2898	0.1060	1.0822	0.066*	
C21	0.18365 (18)	−0.14662 (7)	0.8563 (2)	0.0583 (4)	
H21A	0.2464	−0.1519	0.9552	0.070*	
H21B	0.2156	−0.1184	0.8030	0.070*	
C22	0.1652 (2)	−0.20017 (8)	0.7689 (3)	0.0819 (7)	
H22A	0.1076	−0.1940	0.6674	0.098*	
H22B	0.2466	−0.2123	0.7606	0.098*	
C23	−0.0016 (3)	−0.22429 (8)	0.8599 (3)	0.0983 (8)	
H23A	−0.0344	−0.2532	0.9103	0.118*	
H23B	−0.0641	−0.2179	0.7615	0.118*	
C24	0.0157 (2)	−0.17187 (7)	0.9519 (3)	0.0706 (5)	
H24A	−0.0652	−0.1607	0.9638	0.085*	
H24B	0.0762	−0.1781	1.0518	0.085*	

### Atomic displacement parameters (Å^2^)

**Table:**

	*U*^11^	*U*^22^	*U*^33^	*U*^12^	*U*^13^	*U*^23^
O1	0.0535 (7)	0.0607 (8)	0.0796 (9)	0.0192 (6)	0.0119 (6)	0.0079 (6)
O2	0.1027 (11)	0.0632 (8)	0.0669 (9)	−0.0336 (8)	0.0078 (8)	−0.0112 (7)
O3	0.1412 (15)	0.0329 (7)	0.1057 (12)	0.0187 (8)	0.0165 (11)	0.0031 (7)
N1	0.0403 (7)	0.0374 (6)	0.0435 (7)	0.0050 (5)	0.0026 (5)	0.0000 (5)
N2	0.0441 (7)	0.0375 (6)	0.0465 (7)	−0.0053 (5)	0.0100 (6)	−0.0001 (5)
N3	0.0563 (8)	0.0282 (6)	0.0533 (8)	0.0020 (5)	0.0077 (6)	0.0027 (5)
C1	0.0403 (8)	0.0338 (7)	0.0366 (7)	0.0064 (6)	0.0084 (6)	−0.0055 (6)
C2	0.0435 (8)	0.0328 (7)	0.0367 (7)	0.0034 (6)	0.0106 (6)	−0.0026 (6)
C3	0.0401 (8)	0.0313 (7)	0.0394 (8)	0.0018 (6)	0.0107 (6)	−0.0053 (6)
C4	0.0409 (8)	0.0314 (7)	0.0410 (8)	0.0046 (6)	0.0074 (6)	−0.0037 (6)
C5	0.0453 (8)	0.0274 (7)	0.0401 (8)	0.0033 (6)	0.0117 (6)	−0.0038 (6)
C6	0.0388 (8)	0.0313 (7)	0.0436 (8)	0.0024 (6)	0.0108 (6)	−0.0076 (6)
C7	0.0450 (8)	0.0455 (8)	0.0384 (8)	0.0043 (6)	0.0047 (6)	−0.0054 (6)
C8	0.0579 (10)	0.0461 (9)	0.0549 (10)	0.0020 (7)	0.0116 (8)	0.0113 (7)
C9	0.0418 (8)	0.0413 (8)	0.0480 (9)	0.0017 (6)	0.0124 (7)	−0.0014 (6)
C10	0.0475 (9)	0.0420 (8)	0.0644 (11)	0.0031 (7)	0.0010 (8)	0.0068 (8)
C11	0.0560 (9)	0.0341 (8)	0.0470 (9)	0.0000 (6)	0.0135 (7)	0.0000 (6)
C12	0.0465 (9)	0.0391 (8)	0.0721 (11)	−0.0027 (7)	0.0121 (8)	−0.0037 (8)
C13	0.0426 (9)	0.0508 (9)	0.0622 (11)	−0.0019 (7)	−0.0003 (8)	−0.0053 (8)
C14	0.0437 (10)	0.0677 (12)	0.0866 (14)	−0.0008 (8)	0.0086 (9)	0.0056 (10)
C15	0.0647 (11)	0.0527 (10)	0.0638 (11)	0.0154 (8)	0.0155 (9)	−0.0023 (8)
C16	0.0492 (9)	0.0409 (8)	0.0482 (9)	0.0018 (7)	0.0065 (7)	−0.0026 (7)
C17	0.0642 (11)	0.0580 (10)	0.0578 (10)	−0.0192 (8)	0.0148 (9)	0.0045 (8)
C18	0.1019 (16)	0.0556 (11)	0.0723 (13)	−0.0325 (11)	0.0117 (12)	0.0004 (10)
C19	0.1005 (16)	0.0594 (11)	0.0640 (12)	−0.0135 (11)	0.0257 (11)	−0.0139 (9)
C20	0.0662 (11)	0.0467 (9)	0.0513 (9)	−0.0085 (8)	0.0183 (8)	−0.0014 (7)
C21	0.0679 (11)	0.0450 (9)	0.0562 (10)	0.0109 (8)	0.0113 (9)	0.0005 (8)
C22	0.1139 (18)	0.0472 (11)	0.0763 (14)	0.0241 (11)	0.0178 (13)	−0.0038 (10)
C23	0.120 (2)	0.0357 (10)	0.125 (2)	−0.0137 (12)	0.0169 (17)	0.0026 (11)
C24	0.0800 (13)	0.0394 (9)	0.0888 (14)	−0.0077 (9)	0.0214 (11)	0.0096 (9)

### Geometric parameters (Å, °)

**Table:**

O1—C14	1.411 (2)	C10—H10E	0.9600
O1—C15	1.423 (2)	C10—H10F	0.9600
O2—C19	1.410 (2)	C11—H11A	0.9700
O2—C18	1.424 (2)	C11—H11B	0.9700
O3—C23	1.415 (3)	C12—H12A	0.9600
O3—C22	1.420 (3)	C12—H12B	0.9600
N1—C16	1.4555 (19)	C12—H12C	0.9600
N1—C13	1.4612 (19)	C12—H12D	0.9600
N1—C7	1.4669 (19)	C12—H12E	0.9600
N2—C20	1.453 (2)	C12—H12F	0.9600
N2—C17	1.464 (2)	C13—C14	1.511 (3)
N2—C9	1.4670 (19)	C13—H13A	0.9700
N3—C21	1.458 (2)	C13—H13B	0.9700
N3—C24	1.460 (2)	C14—H14A	0.9700
N3—C11	1.4676 (19)	C14—H14B	0.9700
C1—C6	1.404 (2)	C15—C16	1.505 (2)
C1—C2	1.404 (2)	C15—H15A	0.9700
C1—C7	1.5241 (19)	C15—H15B	0.9700
C2—C3	1.408 (2)	C16—H16A	0.9700
C2—C8	1.515 (2)	C16—H16B	0.9700
C3—C4	1.403 (2)	C17—C18	1.500 (3)
C3—C9	1.523 (2)	C17—H17A	0.9700
C4—C5	1.409 (2)	C17—H17B	0.9700
C4—C10	1.518 (2)	C18—H18A	0.9700
C5—C6	1.4054 (19)	C18—H18B	0.9700
C5—C11	1.520 (2)	C19—C20	1.507 (2)
C6—C12	1.511 (2)	C19—H19A	0.9700
C7—H7A	0.9700	C19—H19B	0.9700
C7—H7B	0.9700	C20—H20A	0.9700
C8—H8A	0.9600	C20—H20B	0.9700
C8—H8B	0.9600	C21—C22	1.506 (2)
C8—H8C	0.9600	C21—H21A	0.9700
C8—H8D	0.9600	C21—H21B	0.9700
C8—H8E	0.9600	C22—H22A	0.9700
C8—H8F	0.9600	C22—H22B	0.9700
C9—H9A	0.9700	C23—C24	1.505 (3)
C9—H9B	0.9700	C23—H23A	0.9700
C10—H10A	0.9600	C23—H23B	0.9700
C10—H10B	0.9600	C24—H24A	0.9700
C10—H10C	0.9600	C24—H24B	0.9700
C10—H10D	0.9600		
			
C14—O1—C15	109.82 (13)	H12A—C12—H12B	109.5
C19—O2—C18	109.45 (15)	C6—C12—H12C	109.5
C23—O3—C22	110.11 (16)	H12A—C12—H12C	109.5
C16—N1—C13	108.21 (12)	H12B—C12—H12C	109.5
C16—N1—C7	112.33 (12)	C6—C12—H12D	109.5
C13—N1—C7	111.20 (12)	H12A—C12—H12D	141.1
C20—N2—C17	108.33 (13)	H12B—C12—H12D	56.3
C20—N2—C9	112.00 (12)	H12C—C12—H12D	56.3
C17—N2—C9	110.53 (13)	C6—C12—H12E	109.5
C21—N3—C24	108.54 (13)	H12A—C12—H12E	56.3
C21—N3—C11	112.74 (13)	H12B—C12—H12E	141.1
C24—N3—C11	111.04 (13)	H12C—C12—H12E	56.3
C6—C1—C2	119.84 (13)	H12D—C12—H12E	109.5
C6—C1—C7	118.80 (13)	C6—C12—H12F	109.5
C2—C1—C7	121.22 (13)	H12A—C12—H12F	56.3
C1—C2—C3	119.75 (13)	H12B—C12—H12F	56.3
C1—C2—C8	120.73 (13)	H12C—C12—H12F	141.1
C3—C2—C8	119.52 (13)	H12D—C12—H12F	109.5
C4—C3—C2	120.40 (13)	H12E—C12—H12F	109.5
C4—C3—C9	121.99 (13)	N1—C13—C14	108.98 (15)
C2—C3—C9	117.52 (13)	N1—C13—H13A	109.9
C3—C4—C5	119.64 (13)	C14—C13—H13A	109.9
C3—C4—C10	120.48 (13)	N1—C13—H13B	109.9
C5—C4—C10	119.87 (13)	C14—C13—H13B	109.9
C6—C5—C4	119.85 (13)	H13A—C13—H13B	108.3
C6—C5—C11	118.19 (13)	O1—C14—C13	111.93 (15)
C4—C5—C11	121.94 (13)	O1—C14—H14A	109.2
C1—C6—C5	120.29 (13)	C13—C14—H14A	109.2
C1—C6—C12	119.83 (13)	O1—C14—H14B	109.2
C5—C6—C12	119.87 (13)	C13—C14—H14B	109.2
N1—C7—C1	114.26 (12)	H14A—C14—H14B	107.9
N1—C7—H7A	108.7	O1—C15—C16	111.83 (15)
C1—C7—H7A	108.7	O1—C15—H15A	109.2
N1—C7—H7B	108.7	C16—C15—H15A	109.2
C1—C7—H7B	108.7	O1—C15—H15B	109.2
H7A—C7—H7B	107.6	C16—C15—H15B	109.2
C2—C8—H8A	109.5	H15A—C15—H15B	107.9
C2—C8—H8B	109.5	N1—C16—C15	110.03 (14)
H8A—C8—H8B	109.5	N1—C16—H16A	109.7
C2—C8—H8C	109.5	C15—C16—H16A	109.7
H8A—C8—H8C	109.5	N1—C16—H16B	109.7
H8B—C8—H8C	109.5	C15—C16—H16B	109.7
C2—C8—H8D	109.5	H16A—C16—H16B	108.2
H8A—C8—H8D	141.1	N2—C17—C18	110.69 (16)
H8B—C8—H8D	56.3	N2—C17—H17A	109.5
H8C—C8—H8D	56.3	C18—C17—H17A	109.5
C2—C8—H8E	109.5	N2—C17—H17B	109.5
H8A—C8—H8E	56.3	C18—C17—H17B	109.5
H8B—C8—H8E	141.1	H17A—C17—H17B	108.1
H8C—C8—H8E	56.3	O2—C18—C17	111.44 (16)
H8D—C8—H8E	109.5	O2—C18—H18A	109.3
C2—C8—H8F	109.5	C17—C18—H18A	109.3
H8A—C8—H8F	56.3	O2—C18—H18B	109.3
H8B—C8—H8F	56.3	C17—C18—H18B	109.3
H8C—C8—H8F	141.1	H18A—C18—H18B	108.0
H8D—C8—H8F	109.5	O2—C19—C20	111.81 (17)
H8E—C8—H8F	109.5	O2—C19—H19A	109.3
N2—C9—C3	114.27 (12)	C20—C19—H19A	109.3
N2—C9—H9A	108.7	O2—C19—H19B	109.3
C3—C9—H9A	108.7	C20—C19—H19B	109.3
N2—C9—H9B	108.7	H19A—C19—H19B	107.9
C3—C9—H9B	108.7	N2—C20—C19	110.91 (14)
H9A—C9—H9B	107.6	N2—C20—H20A	109.5
C4—C10—H10A	109.5	C19—C20—H20A	109.5
C4—C10—H10B	109.5	N2—C20—H20B	109.5
H10A—C10—H10B	109.5	C19—C20—H20B	109.5
C4—C10—H10C	109.5	H20A—C20—H20B	108.0
H10A—C10—H10C	109.5	N3—C21—C22	110.32 (16)
H10B—C10—H10C	109.5	N3—C21—H21A	109.6
C4—C10—H10D	109.5	C22—C21—H21A	109.6
H10A—C10—H10D	141.1	N3—C21—H21B	109.6
H10B—C10—H10D	56.3	C22—C21—H21B	109.6
H10C—C10—H10D	56.3	H21A—C21—H21B	108.1
C4—C10—H10E	109.5	O3—C22—C21	111.68 (18)
H10A—C10—H10E	56.3	O3—C22—H22A	109.3
H10B—C10—H10E	141.1	C21—C22—H22A	109.3
H10C—C10—H10E	56.3	O3—C22—H22B	109.3
H10D—C10—H10E	109.5	C21—C22—H22B	109.3
C4—C10—H10F	109.5	H22A—C22—H22B	107.9
H10A—C10—H10F	56.3	O3—C23—C24	111.9 (2)
H10B—C10—H10F	56.3	O3—C23—H23A	109.2
H10C—C10—H10F	141.1	C24—C23—H23A	109.2
H10D—C10—H10F	109.5	O3—C23—H23B	109.2
H10E—C10—H10F	109.5	C24—C23—H23B	109.2
N3—C11—C5	113.58 (12)	H23A—C23—H23B	107.9
N3—C11—H11A	108.8	N3—C24—C23	108.98 (18)
C5—C11—H11A	108.8	N3—C24—H24A	109.9
N3—C11—H11B	108.8	C23—C24—H24A	109.9
C5—C11—H11B	108.8	N3—C24—H24B	109.9
H11A—C11—H11B	107.7	C23—C24—H24B	109.9
C6—C12—H12A	109.5	H24A—C24—H24B	108.3
C6—C12—H12B	109.5		
			
C6—C1—C2—C3	5.1 (2)	C4—C3—C9—N2	−105.37 (15)
C7—C1—C2—C3	−170.54 (13)	C2—C3—C9—N2	77.97 (16)
C6—C1—C2—C8	−175.31 (13)	C21—N3—C11—C5	75.51 (16)
C7—C1—C2—C8	9.0 (2)	C24—N3—C11—C5	−162.43 (14)
C1—C2—C3—C4	−3.6 (2)	C6—C5—C11—N3	72.68 (17)
C8—C2—C3—C4	176.80 (13)	C4—C5—C11—N3	−108.64 (15)
C1—C2—C3—C9	173.10 (12)	C16—N1—C13—C14	−59.13 (18)
C8—C2—C3—C9	−6.48 (19)	C7—N1—C13—C14	177.05 (14)
C2—C3—C4—C5	−0.8 (2)	C15—O1—C14—C13	−57.6 (2)
C9—C3—C4—C5	−177.38 (13)	N1—C13—C14—O1	59.8 (2)
C2—C3—C4—C10	177.94 (14)	C14—O1—C15—C16	56.5 (2)
C9—C3—C4—C10	1.4 (2)	C13—N1—C16—C15	58.77 (17)
C3—C4—C5—C6	3.7 (2)	C7—N1—C16—C15	−178.10 (12)
C10—C4—C5—C6	−175.03 (14)	O1—C15—C16—N1	−58.12 (18)
C3—C4—C5—C11	−174.93 (12)	C20—N2—C17—C18	−56.39 (19)
C10—C4—C5—C11	6.3 (2)	C9—N2—C17—C18	−179.45 (14)
C2—C1—C6—C5	−2.2 (2)	C19—O2—C18—C17	−58.3 (2)
C7—C1—C6—C5	173.55 (12)	N2—C17—C18—O2	58.7 (2)
C2—C1—C6—C12	177.06 (13)	C18—O2—C19—C20	57.9 (2)
C7—C1—C6—C12	−7.2 (2)	C17—N2—C20—C19	55.92 (19)
C4—C5—C6—C1	−2.2 (2)	C9—N2—C20—C19	178.08 (15)
C11—C5—C6—C1	176.48 (12)	O2—C19—C20—N2	−58.3 (2)
C4—C5—C6—C12	178.50 (13)	C24—N3—C21—C22	58.08 (19)
C11—C5—C6—C12	−2.8 (2)	C11—N3—C21—C22	−178.46 (14)
C16—N1—C7—C1	79.92 (16)	C23—O3—C22—C21	56.2 (2)
C13—N1—C7—C1	−158.63 (13)	N3—C21—C22—O3	−57.3 (2)
C6—C1—C7—N1	71.62 (16)	C22—O3—C23—C24	−57.9 (3)
C2—C1—C7—N1	−112.69 (15)	C21—N3—C24—C23	−58.8 (2)
C20—N2—C9—C3	73.86 (16)	C11—N3—C24—C23	176.75 (17)
C17—N2—C9—C3	−165.24 (13)	O3—C23—C24—N3	59.8 (2)

### Hydrogen-bond geometry (Å, °)

**Table:**

*D*—H···*A*	*D*—H	H···*A*	*D*···*A*	*D*—H···*A*
C11—H11B···Cg1^i^	0.97	2.90	3.731 (2)	144
C7—H7B···Cg1^ii^	0.97	2.80	3.528 (2)	132

Symmetry codes: (i) −*x*, −*y*, −*z*+2; (ii) −*x*, −*y*, −*z*+1.
